# TAM receptors, Phosphatidylserine, inflammation, and Cancer

**DOI:** 10.1186/s12964-019-0461-0

**Published:** 2019-11-27

**Authors:** Tal Burstyn-Cohen, Avi Maimon

**Affiliations:** Institute for Dental Sciences, Faculty of Dental Medicine, The Hebrew University-Hadassah, Jerusalem, Israel

**Keywords:** Phosphatidylserine, PtdSer, TAM receptors, Protein S, PROS1, GAS6, Cancer, Inflammation

## Abstract

**Abstract:**

The numerous and diverse biological roles of Phosphatidylserine (PtdSer) are featured in this special issue. This review will focus on PtdSer as a cofactor required for stimulating TYRO3, AXL and MERTK – comprising the TAM family of receptor tyrosine kinases by their ligands Protein S (PROS1) and growth-arrest-specific 6 (GAS6) in inflammation and cancer. As PtdSer binding to TAMs is a requirement for their activation, the biological repertoire of PtdSer is now recognized to be broadened to include functions performed by TAMs. These include key homeostatic roles necessary for preserving a healthy steady state in different tissues, controlling inflammation and further additional roles in diseased states and cancer. The impact of PtdSer on inflammation and cancer through TAM signaling is a highly dynamic field of research. This review will focus on PtdSer as a necessary component of the TAM receptor-ligand complex, and for maximal TAM signaling. In particular, interactions between tumor cells and their immediate environment - the tumor microenvironment (TME) are highlighted, as both cancer cells and TME express TAMs and secrete their ligands, providing a nexus for a multifold of cross-signaling pathways which affects both immune cells and inflammation as well as tumor cell biology and growth. Here, we will highlight the current and emerging knowledge on the implications of PtdSer on TAM signaling, inflammation and cancer.

**Graphical Abstract:**

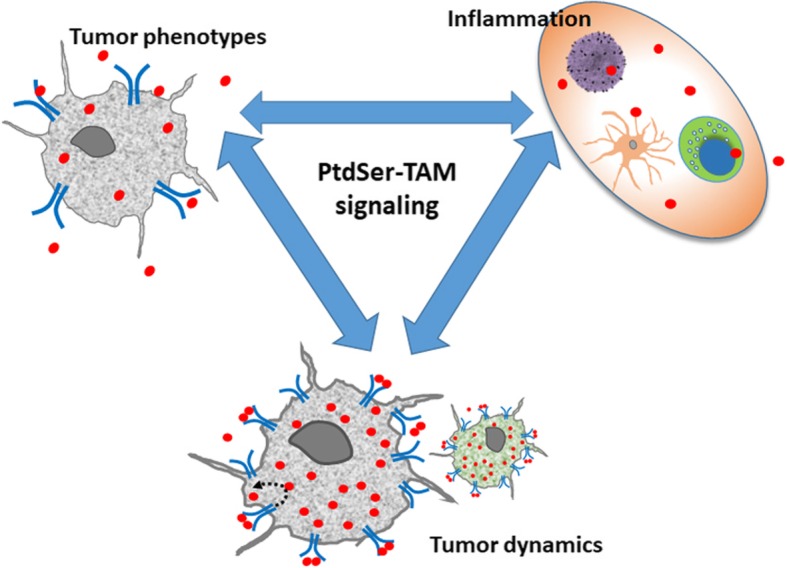

## The elements of TAM signaling and phosphatidylserine (PdtSer)

The core components of the TAM receptor-ligand complex comprise of the three receptors TYRO3, AXL, and MERTK, and two cognate ligands Protein S (PROS1) and Growth-arrest-specific 6 (GAS6). TAMs are ubiquitously expressed by many cell types, and are often co-expressed by various cells – a fact which initially impinged on revealing their roles due to functional redundancy. PROS1 and GAS6 are secreted ligands, which share high structural homology [1–3]. The structure of the three TAM receptors, PROS1 and GAS6, their specific ligand-receptor interactions and binding associations have been extensively described in recent reviews [1–3] and therefore only briefly described below. Their roles in homeostatic regulation are derived from studies where tissue steady state relies both on continuous cell renewal [4, 5] as well as on the rapid clearance of dying and dead cells [6–8] or membranous elements of viable cells [9–13].

PtdSer being a main “eat me” signal marking apoptotic cells (ACs) for clearance on one hand, and TAMs being necessary for AC uptake by phagocytes on the other hand raised the notion that these may be linked. The first physical link between a TAM signaling component and PtdSer was revealed in a 2003 report by Anderson et al. identifying PROS1 as a serum protein which binds to PtdSer. The same work also uncovered the physiological importance of PROS1-PtdSer interaction as responsible for stimulating the phagocytosis of ACs by macrophages [14]. This ability of plasma-borne PROS1 to stimulate efferocytosis (the clearance of apoptotic cells) by macrophages was neither diminished following heat-inactivation of the serum, nor blocked by the intergrin-neutralizing tetrapeptide RGES, indicating PROS1 function was both complement- and integrin-independent, thus may function via another receptor. Moreover, this study also determined that PROS1 binds to PtdSer in a calcium-dependent manner [14].

At that time PROS1 had already been identified as a TAM agonist [15], however its role as an in-vivo genuine TAM agonist was under hot debate [16–20]. Additional reports supporting PROS1 as a TAM ligand appeared several years later in studies investigating the phagocytosis of photoreceptor outer segments by cells of the Retinal Pigment Epithelium (RPE) [13, 21]. The generation of a genetic model allowing the investigation of PROS1 function in different cell types verified its role as a valid TAM agonist [12, 22–25]. Following the identification of GAS6 as a ligand for the TAMs [15, 19] the physiological relevance of GAS6-mediated TAM activation was reported in clearing ACs and in uptake of photoreceptor outer segments [13, 17, 26, 27], which is also PtdSer-dependent.

Both GAS6 and PROS1 exhibit specificity to PtdSer over the other major membrane phospholipids phosphatidylcholine, phosphatidylethanolamine and phosphatidylinositol [14, 28, 29]. Linking the diverse biological functions of TAM receptors to PtdSer through the physical binding of GAS6 and PROS1 constitutes the basis for interactions with membrane-bound TAMs and enables the expansion of the biological repertoire of PtdSer at the same time. The dependence of GAS6 function on PtdSer was reported by Rajotte et al. in 2008, where the interaction between the glutamic acid rich domain (GLA) of GAS6 and PtdSer was shown to be necessary for the survival and intracellular AKT signaling in Human Vascular Endothelial Cells (HUVECs) [30]. More recently, several studies reinforced the emerging concept that while ligand binding to TAM receptors is mostly PtdSer independent, activation of TAMs by these ligands indeed depends on PtdSer. This was demonstrated in a mouse embryonic fibroblast system [25] and in a chimeric reporter cell lines in which the human TAM extracellular and transmembrane domains were fused to the intracellular domain of IFNγR1, where STAT1 phosphorylation was used as a surrogate for TAM activation [31]. Exposure of PtdSer on the surface of T cells is also necessary for the inhibitory effect of T cell-derived PROS1 on dendritic cells (DCs). Carrera-Silva et al. demonstrated that the immunomodulatory effects of PROS1 on DCs were hindered by a physical barrier separating PtdSer from the TAM-receptor expressing DCs, or following Annexin V treatment to mask PtdSer exposure [23]. The presence of PtdSer on ACs also enhances ligand-dependent TAM activation in bone-marrow derived macrophages (BMDMs) [32]. The physiological relevance of PtdSer binding to GLA domains was further extended by Geng et al. demonstrating that in addition to PtdSer exposure in ACs, its exposure by calcium-depleted stressed cells and by tumor-derived exosomal vesicles also function in TAM receptor activation [31].

**Fig. 1:**
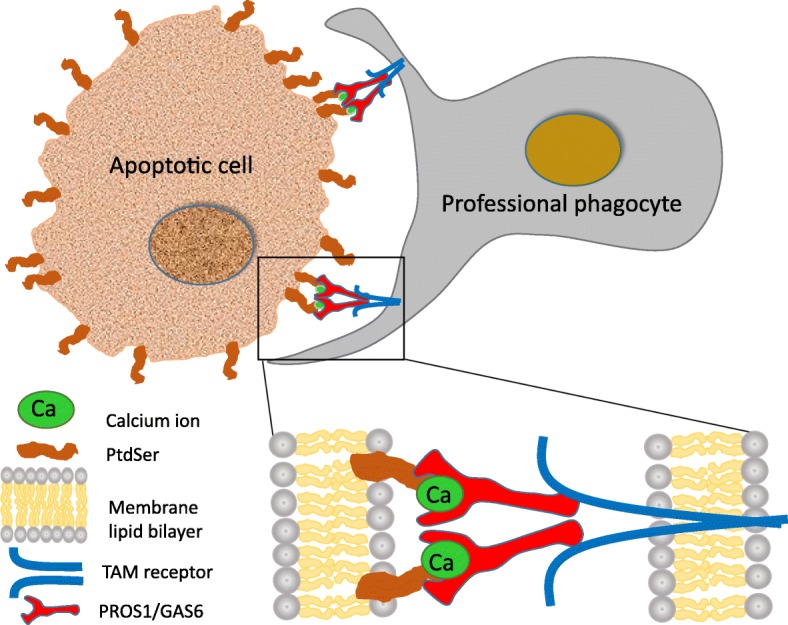
TAM - PtdSer association and the uptake of apoptotic cells. Apoptotic cells (ACs) externalize phosphatidylserine (PtdSer) which is bound by the TAM ligands GAS6 and PROS1. This binding occurs at the GLA domain, present at the amino terminus of the ligands, and is enhanced by the presence of calcium ions, depicted by green ovals. The carboxyl terminal of PROS1 and GAS6 binds to the extracellular domains of the TAM receptors, present on professional phagocytes such as retinal pigment epithelium, Sertoli cells, osteoclasts, macrophages and DCs. TAM receptor activation is optimal in the presence of both ligands and PtdSer. By binding PtdSer on one side and to TAM receptors on the phagocytic cell, PROS1 and GAS6 function as bridging molecules physically linking the phagocyte to the engulfed PtdSer-decorated moiety. In case of macrophage and dendritic cell phagocytes, AC uptake and TAM activation also results in shutting inflammatory signaling and cytokine secretion. Abbreviations: Ca – Calcium ion; PtdSer – phosphatidylserine; TAM – TYRO3, AXL, MERTK, PROS1 – protein S; GAS6 – growth arrest specific 6

The reliance of TAM signaling potential on PtdSer lies within the GLA domains of the ligands PROS1 and GAS6, located at their amino terminus. The vitamin K-dependent gamma carboxylation of the GLA domain dictates the bioactivity of the ligands, as measured by the ability to stimulate TAM receptor phosphorylation [25, 29, 31]. Indeed, Warfarin - an inhibitor of VKORC1, an essential enzyme for the biosynthesis of Vitamin K - may be used to inhibit PROS1 and GAS6 bioactivity [31, 33, 34]. By generating point mutations of key Glutamic acid (Glu) residues of the GLA domain of GAS6, Geng et al. demonstrated these residues directly interact with PtdSer [31]. In order to exert their full bioactivity, the GLA domains of TAM ligands must be complexed with PtdSer through Glu in the presence of calcium ions [25, 29, 31] (Fig. 1). This dependence of PtdSer binding for bioactivity seems to be a broader feature of GLA-containing proteins which interact with cell membranes [35]. In contrast to AXL, activation of both MERTK and TYRO3 by their ligands was enhanced by PtdSer in a concentration-dependent manner, suggesting that local PtdSer concentrations may fine-tune TAM signaling and function [29]. Indeed, membrane PtdSer bound to GAS6 was shown to promote focal (punctate) AXL localization, driving stronger receptor phosphorylation [36]. In conclusion, PtdSer binding to Glu within the GLA domain of PROS1 and GAS6 is indispensable for robust TAM activation, and occurs in the presence of calcium ions. This interaction can occur on any PtdSer-expressing moiety, including apoptotic cells, membrane-derived microparticles (e.g. exosomes), tumor vasculature or viral particles [31, 37–40]. Given the numerous TAM-independent functions of PtdSer [41], and in this special issue, as well as the diverse signaling pathways and cellular functions negotiated by TAMs [1–3, 42], the partnership between PtdSer and TAMs provides a nexus for orchestrating a myriad of membrane-cell biological functions. The influence of the TAM-PtdSer association on inflammation and cancer will be discussed below.

### TAM-PtdSer association in cancer cells

The proto-oncogenic potential of AXL and MERTK was identified immediately upon their cloning from tumor cell lines. Both AXL and MERTK were initially cloned from transformed cells [43–45]. TYRO3 was cloned as a developmental RTK with high expression in the brain and reproductive organs [46–48], and its downstream association with the Src family kinases was subsequently identified [49]. The overexpression of all three receptors in non-malignant cells leads to transformation, inducing tumorigenic features such as increased proliferation and anchorage-independent growth in soft agar [43, 44, 50]. Today, aberrant expression of all three TAM receptors has been documented in a vast number of cancers (reviewed in [42]), stimulating MEK/ERK, PI3K/AKT, JAK/STAT, p38, NFκB and FAK/RAC downstream pathways that provide tumor cells with enhanced proliferative, survival, migratory, invasive and chemo-resistant properties [42, 51, 52]. It is not surprising therefore that overexpression of TAMs is often associated with tumor cell aggressiveness and poor prognosis [51, 53, 54], thus making them attractive targets for therapeutic inactivation, with clinical trials already underway.

To support activation of TAM-dependent oncogenic pathways by PROS1 and GAS6 ligands, PtdSer may be provided by several sources: intra-tumoral apoptotic cells, tumor-associated endothelial cells which were found to be enriched for externalized PtdSer [40], tumor-derived exosomes which are densely coated with exposed PtdSer, or PtdSer exposed by viable tumor cells. Although tumor cells turn on survival pathways to repress apoptosis (including via TAM signaling), ACs are abundant within tumors. Dysregulated protein function, hypoxic foci or chemotherapeutic insult all induce apoptosis, leading to copious local levels of PtdSer within tumors and vasculature. Interestingly, undifferentiated tumor cell lines expose more PtdSer on their outer leaflet compared to their differentiated counterparts [55], suggesting apoptosis as a driver of tumorigenesis through PtdSer signaling. Together, these sources should provide sufficient PtdSer to support TAM oncogenic signaling. It is tempting to speculate that PtdSer exposed by ACs within the tumor may provide cancer cells with the aforementioned TAM-related aggressive characteristics, and at the same time, PtdSer-TAM activation supports the survival of TAM-expressing cancer cells, resulting in clonal selection of those cells with increased aggressiveness. It is therefore likely that PtdSer-linked TAM signaling within tumors provides a mechanism for coupling apoptosis with cell proliferation and enhanced aggressiveness in cancer.

To understand whether TAMs are activated within tumor cells in a ligand-dependent manner, several studies investigated the co-expression of TAM ligands within tumor cells. GAS6 [53, 56–58] and PROS1 [59–61] were both found to be expressed by tumor cells, and led to autocrine activation of the receptors, promoting oncogenic characteristics. Investigating the role of PROS1 in oral squamous cell carcinoma revealed a rather unique mechanism for stimulating oncogenic phenotypes through TAM receptors. In this model, the expression levels of AXL were found to be regulated by PROS1, leading to enhanced cell proliferation and migration. These phenotypes were intercepted in the presence of an AXL-specific inhibitor, indicating the direct involvement of AXL [61]. Thus, Abboud-Jarrous et al. revealed a non-canonical mechanism by which AXL expression and activation is regulated by PROS1 – a TAM ligand which has not been shown to activate AXL through the canonical ligand-receptor interactions [25, 29]. The mechanism by which AXL expression is regulated by PROS1 is still unknown.

Another source of ligand in a tumor setting are the host’s immune cells. Tumor-infiltrating leukocytes were shown to provide the soluble ligand GAS6, which fueled tumor growth and metastatic outcome in several tumor models [62]. A recent study by Zweemer et al. demonstrated the specific contribution of PtdSer (from ACs) to GAS6-mediated AXL activation in triple negative breast cancer and non small cell lung cancer cells, inducing tumor cell migration [63]. Thus, presence of TAM ligands, PtdSer and TAM receptors in tumors allows for pro-tumorigenic PtdSer-TAM signaling, and suggests that targeting either TAM receptors, ligands or PtdSer would similarly lead to reduction in tumor size and improve metastatic load. However, several studies indicate that in reality, TAM receptor-ligand mediated signaling is more complex, especially with respect to the tumor-microenvironment (TME) and inflammation, as discussed below.

### TAM-PtdSer association in the tumor microenvironment: immune modulation and cancer

**Fig. 2:**
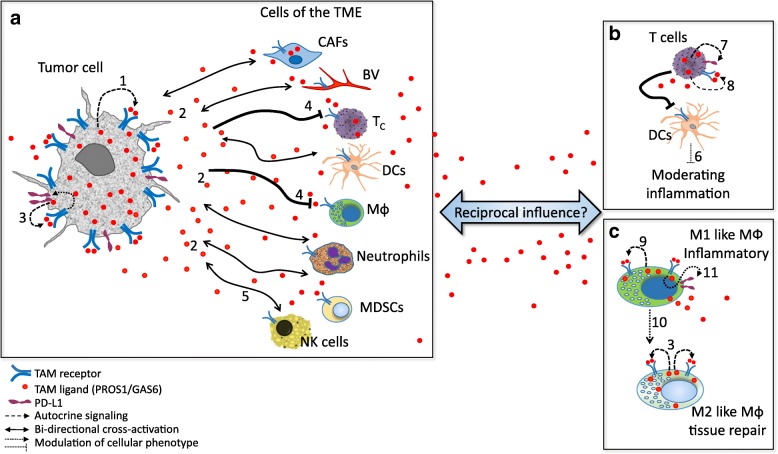
TAM - PtdSer interactions in the tumor microenvironment. Autocrine and paracrine cross-signaling through PtdSer-TAM in a tumor setting. (**a**) Both cancer cells and the different TME cellular compartments express TAM receptors and secrete PROS1 and GAS6. The abundance of PtdSer enables potent autocrine (1) and / or paracrine (2) activation of TAM receptors expressed by tumor cells, resulting in augmented aggressiveness, also by inducing expression of the immune evasion/checkpoint molecules PD-L1 on cancer cells (3, [66]). Tumor-derived TAM ligands suppresses macrophage and T cell infiltration (4, [69, 70]. Similarly, the antitumor cytotoxicity of NK cells is suppressed by TAM receptor expression (5, [33]. (**b**) PtdSer-TAM signaling plays a role in immune cells, where they dampen inflammation, as described for the interactions between T and dendritic cells (6, [23]). Within T cells, opposing roles for TAM signaling report MERTK-dependent signaling to suppresses T cell activation and promote immune evasion through induction of PD-1 expression (7, [71]), but also to provide co-stimulatory functions (8, [72]). (**c**) In the case of macrophages, reports indicate that PstSer-TAM signaling is chiefly anti-inflammatory due to autocrine signaling within M1 and M2-like macrophages (9, [73]), and shifts M1-like pro-inflammatory macrophages towards the anti-inflammatory M2-like state (10, [74]), but also promotes anti-immunity through PD-L1 and PD-L2 expression (11, [71]). Altogether, although PtdSer-TAM signaling may result in opposing outcomes, the net effect of all interactions contributes to the generation of tumors with superior tumorigenic characteristics, within a more permissive environment. See text for details. Abbreviations: CAFs – cancer associated fibroblasts; BV – blood vessel; Tc – T cells; NK – natural killer, MDSCs – myeloid derived suppressor cells; DCs – dendritic cells; MФ – macrophage; TME – tumor microenvironment

**Fig. 3:**
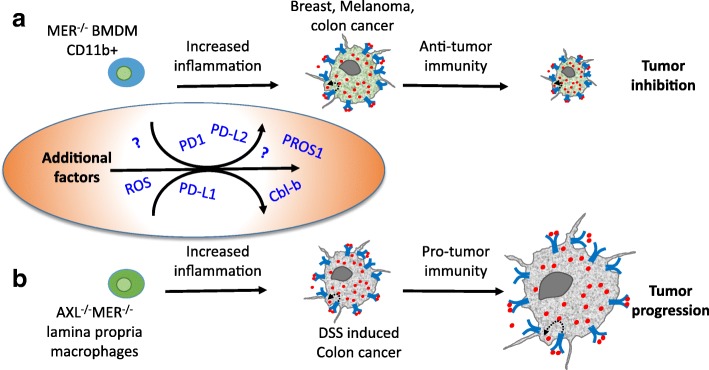
Inhibition of PtdSer-TAM signaling in tumor-immune interactions leads to elevated inflammation but may differentially affect tumor growth. Bone-marrow derived macrophages (BMDMs) differentially influence tumor progression in different cancer models. (**a**) Inhibition of MERTK in CD11b + BMDMs resulted in elevated inflammation, which conveyed anti-tumor immunity resulting in inhibited growth of breast, melanoma and MC38 colon cancer tumors [69]. (**b**) By contrast to (**a**), in a DSS-induced model of colon cancer, the dual inhibition of AXL and MERTK in BMDMs had no effect on tumor progression [76]. Instead, AXL and MERK inhibition in F4/80+; CD11b + lamina propria macrophages conveyed pro-tumor immunity, which promoted cancer progression. These data demonstrate that while inhibition of TAM signaling in macrophages led to inflammation in both cases, opposing effects were imparted on tumor growth, highlighting the complex liaisons between immune and tumor cells through inflammation. Such complexity is likely to be mediated by additional factors, some of which function by immune-modulation, others are yet to be revealed (depicted in the oval). See discussion in the main text

The interaction between tumor cells and host cells comprising their immediate environment greatly affects tumor growth and metastasis [64, 65]. Of particular relevance are immune cells, which are known to interact with and influence tumor progression. Both tumor and immune cells express TAM receptors and secrete their ligands. The scenario where these populations are in great proximity, in a PtdSer rich environment, provides a platform for TAM activation through cross signaling between tumor cells and the host immune cells (Fig. 2). Loges et al. reported that tumor cells educate infiltrating macrophages to upregulate GAS6 expression, which is then secreted and functions as a mitogen for their own growth. Immune cell-derived GAS6 stimulated growth and metastasis of colon, pancreatic, breast and lymphoma cancer models [62]. Interestingly, the growth of melanoma and mammary tumors was significantly inhibited in MERTK^−/−^ host mice, owing to elevated pro-inflammatory (M1-like) cytokine levels in MERTK-deficient CD11b^+^ cells, compared to mice fully expressing MERTK in the host [69]. Increased leukocyte proliferation and higher infiltration of CD8+ T lymphocytes was also observed in the tumors present in MERTK-deficient mice [69]. Thus, MERTK function within the immune compartment of the TME suppresses host antitumor immunity, generating a tumor-supportive milieu [69] (Figs. 2 and 3). Since tumor cells secrete the ligands PROS1 and GAS6, it is hypothesized that they too contribute to this immune suppressive phenotype. Secretion of PROS1 by melanoma cells was shown to skew host macrophages towards the anti-inflammatory M2-like phenotype, in a MERTK and TYRO3-dependent manner, allowing a tumor-permissive environment [70].

Contrary to the above mentioned tumor-suppressive phenotype following MERTK inhibition in CD11b + cells, the inhibition of MERTK and AXL was reported to promote colorectal cancer (CRC) progression [76]. Bosurgi et al. revealed a robust proinflammatory milieu in the lamina propria of AXL^−/−^MERTK^−/−^ mice, which in the case of CRC aggravated tumor growth (Fig. 3). These opposing outcomes following TAM inactivation point to the complexity of TAM signaling and suggests different outcomes in different cancer models. Such disparities may stem from the different impact immune cells have on distinct tumor models, or the diverse functions fulfilled by different ligand-receptor interactions, as a function of differential expression of the TAM repertoire in a particular case. Partnering with other signaling molecules may also underlie such observed functional heterogeneity, as was shown for the AXL - EGFR (Epidermal Growth Factor receptor), which leads to drug resistance in esophageal and Head and Neck cancers [77]. Another factor to consider is that the levels of PtdSer may vary among different tumor environments, which may affect additional, yet unknown factors. This possibility is highlighted by a DSS model of intestinal inflammation, where increased numbers of apoptotic neutrophils were present in the lamina propria of AXL^−/−^MERTK^−/−^ mice, inferring both elevated levels of PtdSer as well as the excessive presence of apoptotic neutrophils both contribute to an increased inflammatory TME in this model of colorectal cancer [76]. The role of PROS1 in immune cells and its impact on tumor progression and metastasis is still unknown, and is currently a subject of active research in our lab.

Another immune-modulatory function driven by TAMs is the upregulation of the immune checkpoint molecule programmed death ligand 1 (PD-L1), promoting the evasion from immune response. Lee-Sherick et al. demonstrated that mice treated with a small molecule MERTK inhibitor not only had decreased numbers of B-ALL leukemia cells in their spleen and bone marrow, but also showed prolonged survival compared to their vehicle-treated control counterparts [71]. Since the leukemia cells used in this study did not express MERTK, Lee-Sherick et al. went on to uncover the MERTK-dependent tumor-suppressive mechanism. Investigating PD-L1 and PD-L2 levels expressed by myeloid cells revealed that MERTK drives PD-L1 and PD-L2 expression on CD11b^+^ monocytes/macrophages and PD-1 expression on T cells in leukemia-bearing mice, contributing to an immunosuppressed milieu, supporting tumor growth [71]. PD-L1 expression driven by TAM receptors was also observed in lung adenocarcinoma and in radiation resistant head and neck carcinoma [66, 67]. Kasikara et al. demonstrated that ectopic TAM expression leads to upregulation of PD-L1 in HEK293 cells. Moreover, basal PD-L1 expression in Hela and MDA-MB-231 breast cancer cells increased following PtdSer-mediated efferocytosis coupled with TAM receptor activation. The TAM-dependent PD-L1 expression was driven by AKT [78]. Furthermore, the study by Kasikara et al. identifies a differential reliance on PtdSer among TAM receptors. TYRO3 and MERTK are considered “PtdSer sensors” as their activation is greatly potentiated in the presence of PtdSer. This is different from AXL, which transduces a strong signal in cancer cells even in the absence of PtdSer [78]. A recent study by Peeters et al. demonstrated that activated human CD8 T cells upregulate PROS1 and MERTK, which function as costimulatory molecules to induce both T cell proliferation and activate cytotoxicity. This in turn supported the expansion of tumor - infiltrating lymphocytes and killing of autologous melanoma cells [72]. Peeters et al. further showed that consumption of soluble PROS1 is high in tumor cells which highly express the TAM receptors, and results in loss of T-cell activation. These results point to a possible competition over PROS1 between tumor and immune cells. However, it remains to be seen whether such competition may alter antitumor immunity. Natural Killer (NK) cells constitute yet another arm of anti-tumor immune defense. In their study, Paolino et al. demonstrated that TAM signaling constitutes an inhibitory pathway for NK cell activation, via Cbl-b [33]. Both Cbl-b ablation and TAM inhibition boosted NK cytotoxicity, leading to decreased melanoma and breast cancer tumors [33]. In conclusion, TAMs and their ligands are expressed both by tumors and by cells of the TME, allowing for bilateral signaling which modulates the immune response, and affects cancer progression. The immune-modulatory role of TAMs stems from their basic anti-inflammatory function in immune cells, which is discussed below.

### TAM-PtdSer association in inflammation

Reviewing the homeostatic roles of PtdSer and TAMs in immune cells at steady state is necessary to fully appreciate their function (as we understand it as of today) in inflammation, apoptotic cell uptake and cancer. Within immune cells, TAMs mediate two important tasks: efferocytosis and constraining the immune response. As potent inhibitors of inflammation, TAMs support the switch towards resolving inflammation and enabling tissue repair. Inactivation of TAM signaling components leads to chronic inflammation and autoimmunity, and has been reviewed extensively [1, 2, 79]. As suppressors of inflammation, activation of TAMs by PtdSer-bound ligands blocks cellular inflammatory signaling through the upregulation of suppressor of cytokine signaling (SOCS) proteins SOCS1 and SOCS3, inactivation of NFκB, and STAT1-dependent shut down of pro-inflammatory cytokine secretion [2, 32, 73, 74, 80]. In the case of phagocytic immune cells, binding of PROS1 and GAS6 to their cognate receptors in the presence of PtdSer induces cytoskeletal changes resulting in the uptake of the PtdSer-coated membrane [25, 29, 32, 52, 74, 78, 81–85]. Failure to clear ACs from tissues often results in toxicity and tissue damage, and boosting AC removal reduces inflammation and ameliorates disease severity [86, 87]. TAM-mediated functions allows macrophages and DCs to maintain steady state by clearing dying and apoptotic cells from tissues [88, 89]. As opposed to engulfment of pathogen-infected cells, there is no interest in mounting an inflammatory immune response upon uptake of non-infected dying or ACs. Thus, the coupling of efferocytosis to anti-inflammatory signaling in immune cells is achieved by the dual role of TAMs.

### Phagocytosis of ACs is anti-inflammatory

The link between phagocytosis of ACs and inhibition of inflammation was discovered in the late 1990’s [90–92]. It should be mentioned that additional PtdSer receptors also mediate phagocytosis and are often co-expressed by different phagocytes, however their particular activation and relative roles is still mostly not understood [88, 93]. Such variability and redundancy highlights phagocytosis as a key biologic function, and is thought to secure distinct modes of phagocytosis under a variety of stimulants and physiologic conditions, providing both resilience and flexibility. Within the TAM family, differential use of TAMs was demonstrated by phagocytes of different types and origin [94], and with respect to the inflammatory stimulus [32]. Successful uptake of a membranous moiety (being it a cell, a viral particle, an extracellular vesicle or a membranous protrusion) by a phagocytic immune cell occurs through the PtdSer exposed on the membrane to be engulfed and a TAM-receptor on the effector immune cell (Fig. 1). The physical link between these membrane bound molecules present on both the engulfing and engulfed sides is provided by the TAM ligands, which serve as bridging molecules: the amino terminus of PROS1 and GAS6 binds to PtdSer and their carboxy end binds to the extracellular domain of TAM receptors, creating the following sequence: externalized PtdSer-TAM ligand-TAM receptor-phagocytic immune cell (Fig. 1).

This bridging by TAM ligands instigates a counter-inflammatory response within phagocytes in immune cells, as was demonstrated for macrophages and DCs [23, 32, 74, 75, 80]. Within macrophages, TAMs promote the shift from the “classical” M1-like pro-inflammatory phenotype, characterized by the secretion of tumor necrosis factor α (TNFα), interleukin (IL) 6, IL-1β, IL-12 and nitric oxide (NO) to the “alternative”, M2-like anti-inflammatory phenotype. The M2-like macrophages are characterized by secretion of the tissue repair promoting cytokines IL-10, IL-13 and transforming growth factor β (TGFβ). By virtue of their anti-inflammatory nature, M2-like macrophages allow a tumor-supportive environment, endorsing tumor progression (Fig. 2).

## Conclusions

Taken together, applying the multiple aspects of TAM-PtdSer biology discussed above into a tumor setting with ample ACs and additional sources of PtdSer, provides a fertile ground for simultaneous cross signaling between cancer and TME cells, both of which express TAMs and secrete PROS1 and GAS6, generating a tumor-supportive environment (Fig. 2). TAMs expressed by tumor-infiltrating macrophages and DCs may be activated either in autocrine or paracrine manners to shut down secretion of inflammatory cytokines and promote a cancer-friendly environment. Tumor-secreted PROS1 modulates host macrophages by shifting them towards the M2-like tissue repair phenotype, facilitating cancer progression. Tumor-infiltrating macrophages secrete GAS6 which supports tumor progression. Secreted TAM agonists bind to TAM receptors overexpressed by cancer cells, to promote oncogenic characteristics and tumor cell aggressiveness (proliferation, migration, cell survival, drug resistance) as well as upregulate PD-L1 expression that promotes immune-evasion. Another level of complexity is supported by the fact that not all tumors respond to inflammation in a similar manner or intensity. Thus, elucidating the complex interactions of PtdSer-TAMs, and their influence on inflammation in a cancer setting would allow to better understand their effect on cancer, and would support the development of advanced anticancer therapies.

## Data Availability

N/A

## Abbreviations

ACs: Apoptotic Cells
BMDMs: Bone-marrow derived macrophages
GLA: Glutamic Acid Rich domain
Glu: Glutamic acid
HUVECs: Human Umbilical Vein Endothelial Cells
IL: Interleukin
NO: Nitric oxide
PD-L1: programmed death ligand 1
TAM: TYRO3, AXL, MERTK receptors
TGFβ: transforming growth factor beta
TME: Tumor microenvironment
